# Lactic acid modified rare earth-based nanomaterials for enhanced radiation therapy by disturbing the glycolysis

**DOI:** 10.1186/s12951-022-01694-1

**Published:** 2022-11-19

**Authors:** Hu Liu, Han Wang, Dalong Ni, Youjia Xu

**Affiliations:** 1 Department of Orthopedics, The Second Affiliated Hospital of Soochow University, Suzhou, 215004 Jiangsu China; 2 Department of Orthopedics, Yancheng Third People’s Hospital, Yancheng, 224001 Jiangsu China; 3 Department of Orthopaedics, Shanghai Key Laboratory for Prevention and Treatment of Bone and Joint Diseases, Shanghai Institute of Traumatology and Orthopaedics, Ruijin Hospital, Shanghai Jiao Tong University School of Medicine, Shanghai, 200025 China

**Keywords:** Nanomedicine, Rare earth, Radiation therapy, Lactic acid, Glycolysis

## Abstract

Deficient deposition of X-rays and strong capacity of repairing damage DNA of cancer cells limit the effect of radiation therapy (RT). Herein, we synthesize CsLu_2_F_7_ nanoparticles with lactic acid (LA) ligands (CsLu_2_F_7_-LA) to overcome these limitations. The high-Z atoms of Lu and Cs can deposit more X-rays for generating enhanced hydroxyl radicals (·OH). Meanwhile, the LA ligand will guide CsLu_2_F_7_-LA to target monocarboxylic acid transporter (MCT) and impede the transportation of free LA, leading to decreased glycolysis and DNA damage repair. Consequently, the curative effect of RT will be enhanced and the strategy of LA accumulation induced radiosensitization is proved by in vivo and in vitro experiments, which will bring prospects for enhanced RT with nanomedicine.

**Supplementary Information:**

The online version contains supplementary material available at 10.1186/s12951-022-01694-1.

## Background

Radiation therapy (RT) is widely used in clinic for the treatments of cancer [1–3]. According to the statistics, nearly 60% of the cancer patients need RT in the treatment plans [4]. In the process of RT, X-ray will decompose water molecules (H_2_O) to generate reactive oxygen species (ROS), mainly hydroxyl radicals (·OH). Generally, ·OH will attack DNA molecules, induce irreversible DNA double-strand breaks (DSBs), and finally lead to the proliferative death of cancer cells [5, 6]. Unfortunately, there are still some obstacles limit the curative effect of RT. Firstly, biological tissues mainly consist of low-Z atoms such as C, H, O, N, P and S, which can deposit few X-rays and result in deficient yield of ·OH [7, 8]. Secondly, cancer cells will devote themselves to repairing the damage caused by RT [9–12]. For example, cancer cells will enhance the metabolism to synthesize enough lipids and proteins to replace the damaged organelles [9, 13]. Hence, it is necessary to develop new methods for increasing the deposition of X-rays and decreasing the metabolism of cancer cells in RT, simultaneously.

Recently, the progress of nanomedicine brings more chances for radiosensitization [14–21]. On the one side, a series of nanomaterials containing high-Z atoms including Lu, Hf, Ta, Au and Bi have been developed for increasing the deposition of X-rays [22–29]. On the other side, the metabolism of cancer cells depends on aerobic glycolysis, which is also called Warburg Effect [30, 31]. Inhibiting the process of glycolysis has much potential for enhancing the effect of cancer therapy [32]. Some small molecules have been developed for inhibiting critical enzymes including hexokinase, pyruvate dehydrogenase and lactate dehydrogenase in the process of glycolysis. In addition, these inhibitors have shown further synergistic effect with RT or chemotherapy [33–36]. Hence, developing high-Z nanomaterials with the ability of disturbing glycolysis will present a satisfactory performance for radiosensitization.

**Fig. 1:**
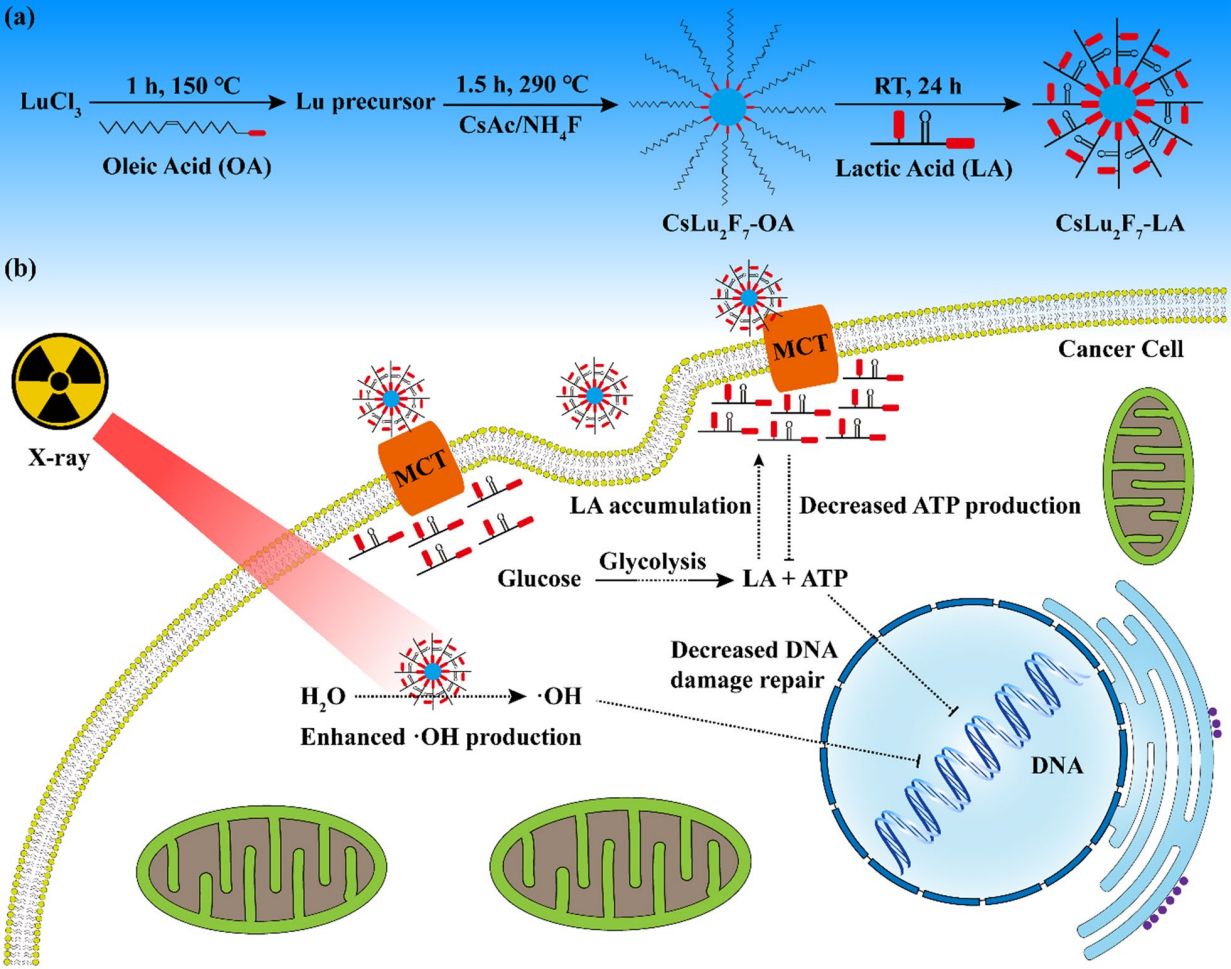
Illustration of **a** synthetic procedure of CsLu_2_F_7_-LA and **b** the mechanism that CsLu_2_F_7_-LA enhance the effect of RT

Lactic acid (LA) is the final product in the process of glycolysis and plays an important role in tumor progression [37]. Cancer cells will generate a mass of LA and excrete them to tumor microenvironment (TME) through monocarboxylic acid transporter (MCT) [38, 39]. The accumulation of LA will decelerate glycolysis and disturb metabolism of cancer cells [38, 39]. Herein, we synthesize CsLu_2_F_7_ nanoparticles with LA ligands (CsLu_2_F_7_-LA) for enhancing the effect of RT. As shown in Fig. 1a, after the synthesis of oleic acid (OA) modified CsLu_2_F_7_ (CsLu_2_F_7_-OA), LA can easily replace OA to obtain water-soluble CsLu_2_F_7_-LA because the strong acid can replace the weak one [40, 41]. As Cs and Lu are high-Z atoms, CsLu_2_F_7_-LA can deposit more X-rays in tumor area (Fig. 1b). In addition, CsLu_2_F_7_-LA will target and disturb the MCT on the cancer cell membrane to block the transportation of LA (Fig. 1b). Then the resultant accumulation of LA will limit the speed of glycolysis and inhibit the repair of DNA DSBs. As a result, the curative effect of RT will be enhanced obviously. We believe this research will bring new ideas and chances for the design of nanomaterials for enhanced RT.

## Materials and methods

### Chemicals and reagents

LuCl_3_, cesium acetate (CsAc), lactic acid (LA), oleic acid (OA), 1-octadecene (ODE) and Rhodamine B (RhB) were purchased from Macklin. NH_4_F, methanol, ethanol and cyclohexane were purchased from Sinopharm Chemical Reagent Co., Ltd. Phosphate buffered solution (PBS), dulbecco’s modified eagle medium (DMEM) and fetal bovine serum (FBS) were obtained from Adamas Life. Cell counting kit-8 (CCK-8), histone H2AX rabbit polyclonal antibody, fluorescein isothiocyanate (FITC), FITC-labeled goat anti-rabbit IgG (H + L), DAPI staining kit, Ki67 staining kit, 2',7'-bis-(2-carboxyethyl)-5-(and-6)-carboxyfluorescein (BCECF) assay kit, 2,7-Dichlorodi-hydrofluorescein diacetate (DCFH-DA) assay kit, ATP assay kit, hematoxylin and eosin (H&E) staining kit, TUNEL apoptosis assay kit, calcein/PI cell viability/cytotoxicity assay kit, Annexin V-FITC/PI apoptosis assay kit were bought from Beyotime.

### Characterization

Transmission electron microscope (TEM) image was carried out by FEI Talos F200X. X-ray diffraction (XRD) was measured by Rigaku D/MAX-2250V. Dynamic light scattering was measured by Brookhaven omni. X-ray photoelectron spectroscopy (XPS) spectra was measured by Thermo Fisher Scientific ESCALAB 250XI. Confocal laser scanning microscopy was carried out on Leica TCS SP8 STED 3X. Fourier transform infrared spectroscopy (FTIR) was measured by Bruker Thermo Fisher Nicolet 6700. The concentration of Lu element was measured by inductively coupled plasma optical emission spectrometer (ICP-OES, Agilent 725). The fluorescence microplate system was TECAN SPARK. Flow Cytometer was BD FACSCalibur. Radiation therapy was carried out by clinical 220 keV X-ray (SARRP, Gulmay Medical Inc.).

### Synthesis of CsLu_2_F_7_-OA and CsLu_2_F_7_-LA

2 mmol LuCl_3_, 20 mL OA and 10 mL ODE were mixed in a flask with three necks and heated to 150 ℃ under the protection of N_2_ for 1 h, and then cooled to room temperature. 7 mmol NH_4_F and 1 mmol CsAc were poured into the flask and stirred for 1 h. Then this mixture was heated to 290 °C for 1.5 h and cooled to room temperature subsequently. After washed with ethanol and cyclohexane for three times, the obtained CsLu_2_F_7_-OA were dispersed in 10 ml cyclohexane. 10 ml as-prepared CsLu_2_F_7_-OA solution in cyclohexane and 10 ml LA solution in water (0.1 M) were mixed and stirred for 4 h. Then this mixture was washed with ethanol for three times, the obtained CsLu_2_F_7_-LA were dispersed in 10 ml water.

### Detection of ·OH in solution

4 ml solution contained RhB (5 ppm) with CsLu_2_F_7_-LA (500 ppm) or water (control group) were placed in centrifuge tubes. These tubes were irradiated with 0 Gy, 5 Gy, 10 Gy, 15 Gy and 20 Gy of X-rays. Supernatants were collected after centrifugation. The ·OH yield was represented by the degradation rate of RhB (absorbance at 554 nm).

### Cells and animals

143B cells (human osteosarcoma cells) and HUEVC (human umbilical vein endothelial cell) cells were purchased from Shanghai Institute of Biochemistry and Cell Biology, Chinese Academy of Sciences. Kunming mice and Balb/c nude mice were purchased from Shanghai SLAC Laboratory Animal Co. Ltd. All the experiments in vivo were approved by the animal ethics committee of Shanghai Jiao Tong University, and the ethics number was 20210918-02.

### Cell viability

143B cells or HUVEC cells (10,000 cells/well) were seeded in the 96-well plates and cultured at 37 °C for 24 h. Next, the medium was replaced by the fresh DMEM medium containing LA and CsLu_2_F_7_-LA at different concentrations and cultured for another 24 h. 100 μl culture media containing 10 μl CCK-8 solution was added and co-incubated with cells for 2 h. The absorbance was measured using a microplate reader at 450 nm.

### Cell apoptosis

143B cells (500,000 cells/well) were seeded in 6-well plates and cultured for 24 h. Then the cells were incubated with DMEM medium and CsLu_2_F_7_-LA (50 ppm) respectively for another 24 h. The cells were irradiated with X-ray (0 Gy or 4 Gy). After 12 h, flow cytometer with Annexin V-FITC/PI double staining was used to evaluate cell apoptosis.

### Detection of DNA DSBs

143B cells (50,000 cells/well) were seeded in confocal dishes (diameter: 20 mm) and cultured for 24 h. Then the cells were incubated with DMEM medium and CsLu_2_F_7_-LA (50 ppm) respectively for another 24 h. The cells were irradiated with X-ray (0 Gy or 4 Gy). After 1 h, the cells were fixed by 4% paraformaldehyde for 15 min and washed three times with PBS. 0.2% Triton X-100 was added to penetrate cells for 10 min. Next, the 1% BSA dissolved in PBS was used to block the cells for another 1 h at room temperature. After incubation with γ-H2AX antibody overnight at 4 °C, the cells were treated with Anti-Rabbit IgG (H + L), F(ab')2 Fragment (Alexa Fluor®488 Conjugate) for 1 h and then stained with DAPI for 15 min. Lastly, the fluorescence of γ-H2AX was observed by a confocal fluorescence microscope.

### Fluorescence imaging of live/dead cells and ROS

143B cells (50,000 cells/well) were seeded in confocal dishes (diameter: 20 mm) and cultured for 24 h. Then the cells were incubated with DMEM medium and CsLu_2_F_7_-LA (50 ppm) respectively for another 24 h. The cells were irradiated with X-ray (4 Gy). After 24 h, the cells were incubated with calcein-AM and PI for 0.5 h. For ROS detection, the cells were incubated with DCFH-DA for 0.5 h. After incubation, the fluorescence was observed by a confocal fluorescence microscope.

### Fluorescence imaging of pH in vitro

143B cells (50,000 cells/well) were seeded in confocal dishes (diameter: 20 mm) and cultured for 24 h. Then the cells were incubated with DMEM medium and CsLu_2_F_7_-LA (50 ppm) respectively for another 24 h. After 24 h, the cells were incubated with BCECF for 0.5 h. After incubation, the fluorescence was observed by a confocal fluorescence microscope.

### Cell clone formation assay

143B cells were seeded in the 6-well plates at various densities of 500, 500, 1000, and 2000 cells/well for 24 h. Then the cells were incubated with DMEM medium and CsLu_2_F_7_-LA (50 ppm) for another 24 h and subsequently irradiated with 0 Gy, 2 Gy, 4 Gy, 6 Gy X-rays. After being cultured with fresh medium for 10 days, the cells were fixed with absolute methyl alcohol and stained with hematoxylin and eosin. The number of colonies was counted by Image-J.

### In vivo biocompatibility assay

The standard H&E staining and blood parameter were conducted to monitor the biocompatibility of CsLu_2_F_7_-LA. The Kunming mice (7 weeks, female) were injected with CsLu_2_F_7_-LA (0–80 mg/kg) through the tail vein. The main tissues (heart, liver, spleen, lung and kidney) of Kunming mice were dissected for H&E staining at 30 days post-injection. The blood of Kunming mice was harvested for blood routine test and biochemical examination.

### In vivo radiation therapy

To set up the xenograft tumor model, 143B cells (1 × 10^6^ cells) were injected subcutaneously into Balb/c nude mice (7 weeks, female). When the tumor volume reached about 100 cm^3^, the mice were divided four groups randomly: (i) Control, (ii) CsLu_2_F_7_-LA, (iii) Control + X-ray, (iv) CsLu_2_F_7_-LA + X-ray. PBS (10 μL) and CsLu_2_F_7_-LA (1 mg, 10 μL) were injected into tumors directly. After 24 h, the tumors of groups iii and iv were irradiated with X-rays (6 Gy). After 48 h, the H&E, TUNEL and Ki67 staining of the tumor tissues were performed by commercially available kits. The body weight and tumor volume of the mice were measured every 2 days.

## Results and discussion

### Synthesis and characterization of CsLu_2_F_7_-LA

**Fig. 2:**
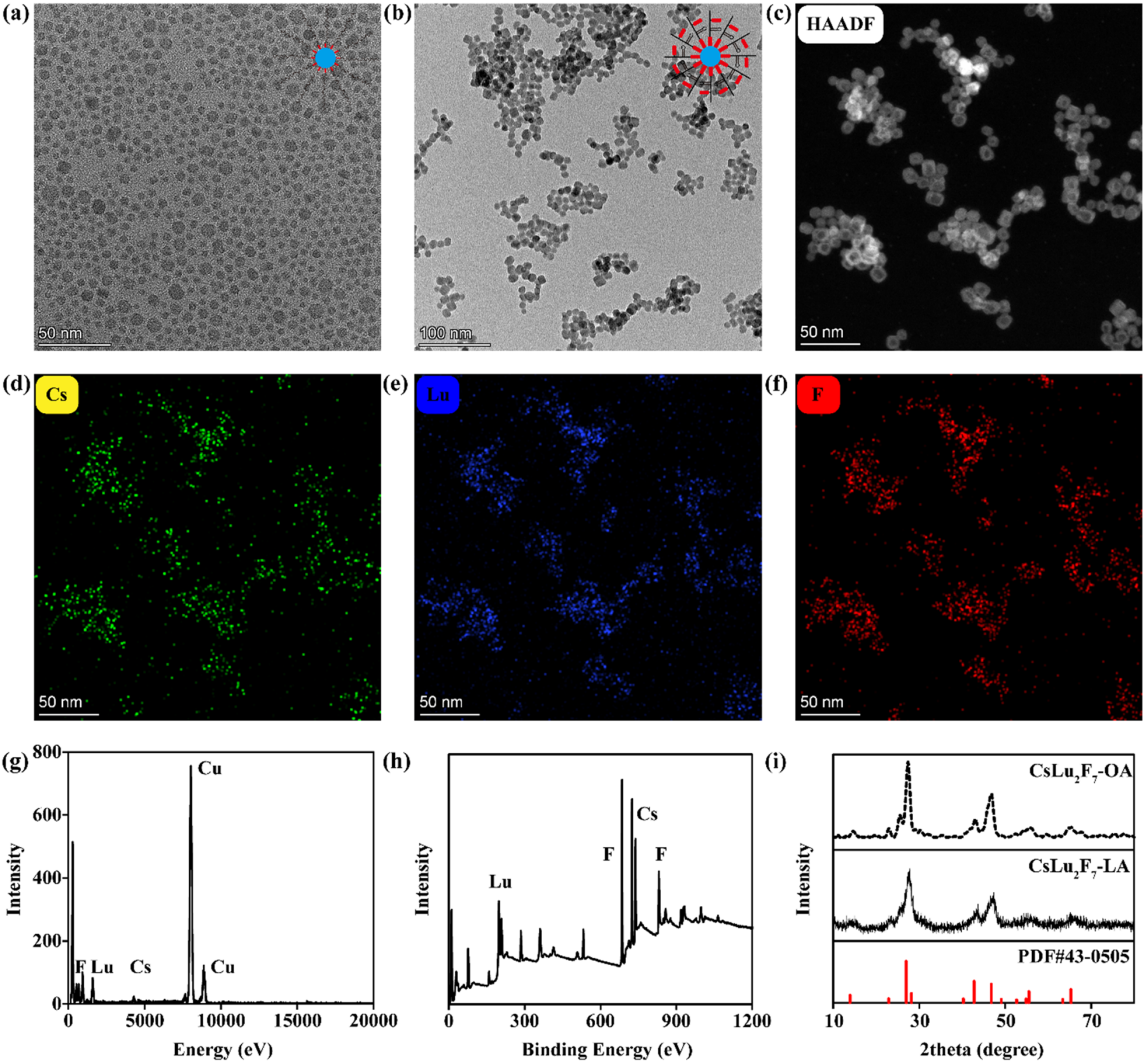
Characterization of nanomaterials. **a** TEM image of CsLu_2_F_7_-OA. **b** TEM image of CsLu_2_F_7_-LA. **c** HAADF image of CsLu_2_F_7_-LA. **d** Cs, **e** Lu and **f** F Elements mapping of CsLu_2_F_7_-LA. **g** EDS spectrum of CsLu_2_F_7_-LA. **h** XPS spectrum of CsLu_2_F_7_-LA. **i** XRD pattern of CsLu_2_F_7_-OA and CsLu_2_F_7_-LA

As shown in Fig. 1a, CsLu_2_F_7_-LA are synthesized by the pyrolysis [42, 43]. We first use LuCl_3_, OA and octadecene (ODE) to react at 150 ℃ to obtain the Lu precursor (lutecium oleate). Then this precursor is mixed with cesium acetate (CsAc) and ammonium fluoride (NH_4_F), and react at 290 ℃ to obtain CsLu_2_F_7_-OA. To obtain CsLu_2_F_7_-LA, the CsLu_2_F_7_-OA cyclohexane solution is mixed with LA water solution for 24 h, during this process LA will gradually replace OA. As shown in Fig. 2a, transmission electron microscopy (TEM) image shows that CsLu_2_F_7_-OA is monodispersed with a size of ~ 10 nm, while the hydrodynamic radius of CsLu_2_F_7_-OA (Additional file 1: Fig. S1) measured by dynamic light scattering (DLS) is 12.7 nm. The Fourier tansform infrared (FTIR) spectra of CsLu_2_F_7_-OA (Additional file 1: Fig. S2) presents the strong absorption of methylene asymmetric carbon-hydrogen bonds (C-H) stretching and methylene symmetric C-H stretching, which come from OA molecules [44]. In contrast, CsLu_2_F_7_-LA has little absorption of C-H stretching, which proves the successful modification of LA. After LA modification, CsLu_2_F_7_-LA present slight agglomeration (Fig. 2b), but still remain stable in deionized (DI) water, saline and dulbecco's modified eagle medium (DMEM) (Additional file 1: Fig. S3). The high angle annular dark field-scanning transmission electron microscopy (HAADF-STEM) image (Fig. 2c) and elemental mapping (Fig. 2d–f) show the existence of Cs atoms, Lu atoms, and F atoms in CsLu_2_F_7_-LA. In addition, the data of energy disperse spectroscopy (EDS) and X-ray photoelectron spectroscopy (XPS) also show that CsLu_2_F_7_-LA contain Cs, Lu and F elements (Fig. 2g, h and Additional file 1: Fig. S4). Finally, X-Ray diffraction (XRD) pattern shows that the crystal structures of CsLu_2_F_7_-OA and CsLu_2_F_7_-LA are in accordance with standrad sample (Fig. 2i), indicating that the LA modification does not change the crystal structure of CsLu_2_F_7_. The yield of ·OH in solutions is detected by the degradation of Rhodamine B (RhB). As shown in Additional file 1: Fig. S5, CsLu_2_F_7_-LA can induce more ·OH than control group (DI water), presenting that CsLu_2_F_7_-LA can deposit more X-rays.

### CsLu_2_F_7_-LA impede the glycolysis in vitro

**Fig. 3:**
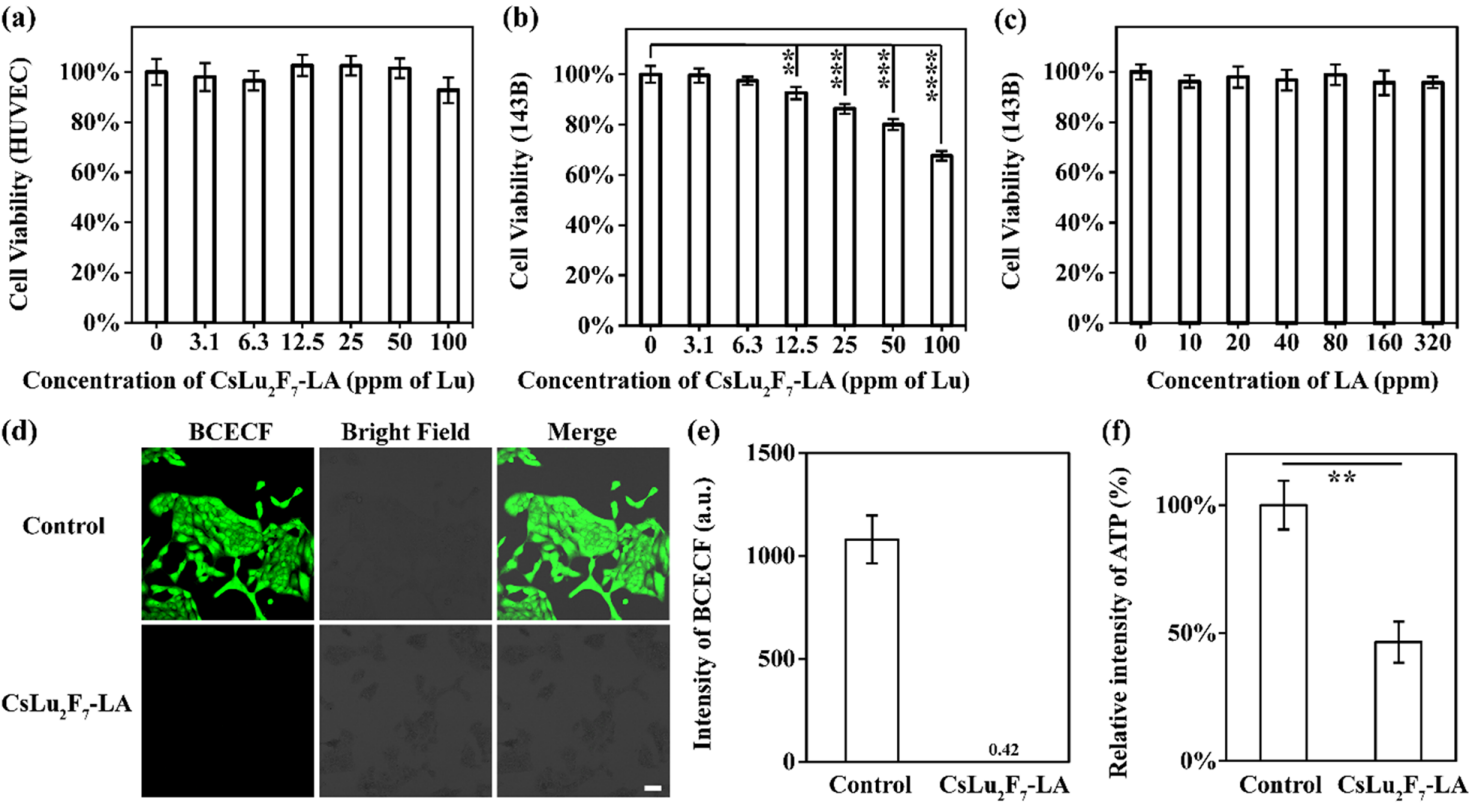
Experiments in vitro. **a** 143B cells viability of different concentrations of LA (n = 6, mean ± SD). **b** HUVEC cells viability of different concentrations of CsLu_2_F_7_-LA (n = 6, mean ± SD). **c** 143B cells viability of different concentrations of CsLu_2_F_7_-LA (n = 6, mean ± SD). **d** 143B cell pH staining of control group and CsLu_2_F_7_-LA group. Scale bar: 50 μm. **e** Relative intensity of BCECF in control group and CsLu_2_F_7_-LA group (n = 3, mean ± SD). **f** Relative content of ATP in control group and CsLu_2_F_7_-LA group (n = 3, mean ± SD). Two asterisks indicate P < 0.01, three asterisks indicate P < 0.001, and four asterisks indicate P < 0.0001 according to Student’s two-tailed t-test

After the synthesis and characterization of CsLu_2_F_7_-LA, we clarify if they could disturb the glycolysis through blocking MCT. As shown in Fig. 3a, CsLu_2_F_7_-LA present little cytotoxicity to human umbilical vein endothelial cell (HUVEC) even at the concentration of 100 ppm (Lu atoms). While as shown in Fig. 3b, the same dosage of CsLu_2_F_7_-LA makes obvious damage to 143B cells (human osteosarcoma cell). LA is a kind of molecule that exists in the human body, and present little cytotoxicity to 143B cells (Fig. 3c). In addition, it is worth noting that there are few free ions release from CsLu_2_F_7_-LA (Additional file 1: Fig. S6). Hence, we suppose that the cytotoxicity of CsLu_2_F_7_-LA to 143B cells can be attributed to the blocking of MCT. To verify this hypothesis, we detected the pH of 143B cells by 2',7'-bis-(2-carboxyethyl)-5-(and-6)-carboxyfluorescein (BCECF) staining. If CsLu_2_F_7_-LA block MCT, LA will accumulate in cells and the pH will decrease. As shown in Fig. 3d, e the fluorescence of BCECF of CsLu_2_F_7_-LA group is lower than that of control group, indicating CsLu_2_F_7_-LA can decrease the pH of 143B cells. Additionally, as shown in Fig. 3f, the decreased content of adenosine triphosphate (ATP) of CsLu_2_F_7_-LA group further prove that the accumulation of LA will impede the process of glycolysis. The cytophagy of CsLu_2_F_7_-LA is detected by confocal microscopy. CsLu_2_F_7_-LA are decorated with fluorescein isothiocyanate (FITC) through electrostatic adsorption. After co-culture for 4 h, the fluorescence of FITC is found in 143B cells, indicating that CsLu_2_F_7_-LA can enter into cells (Additional file 1: Fig. S7). All these data show that CsLu_2_F_7_-LA can impede glycolysis through blocking MCT.

### CsLu_2_F_7_-LA enhance the effect of RT in vitro

**Fig. 4:**
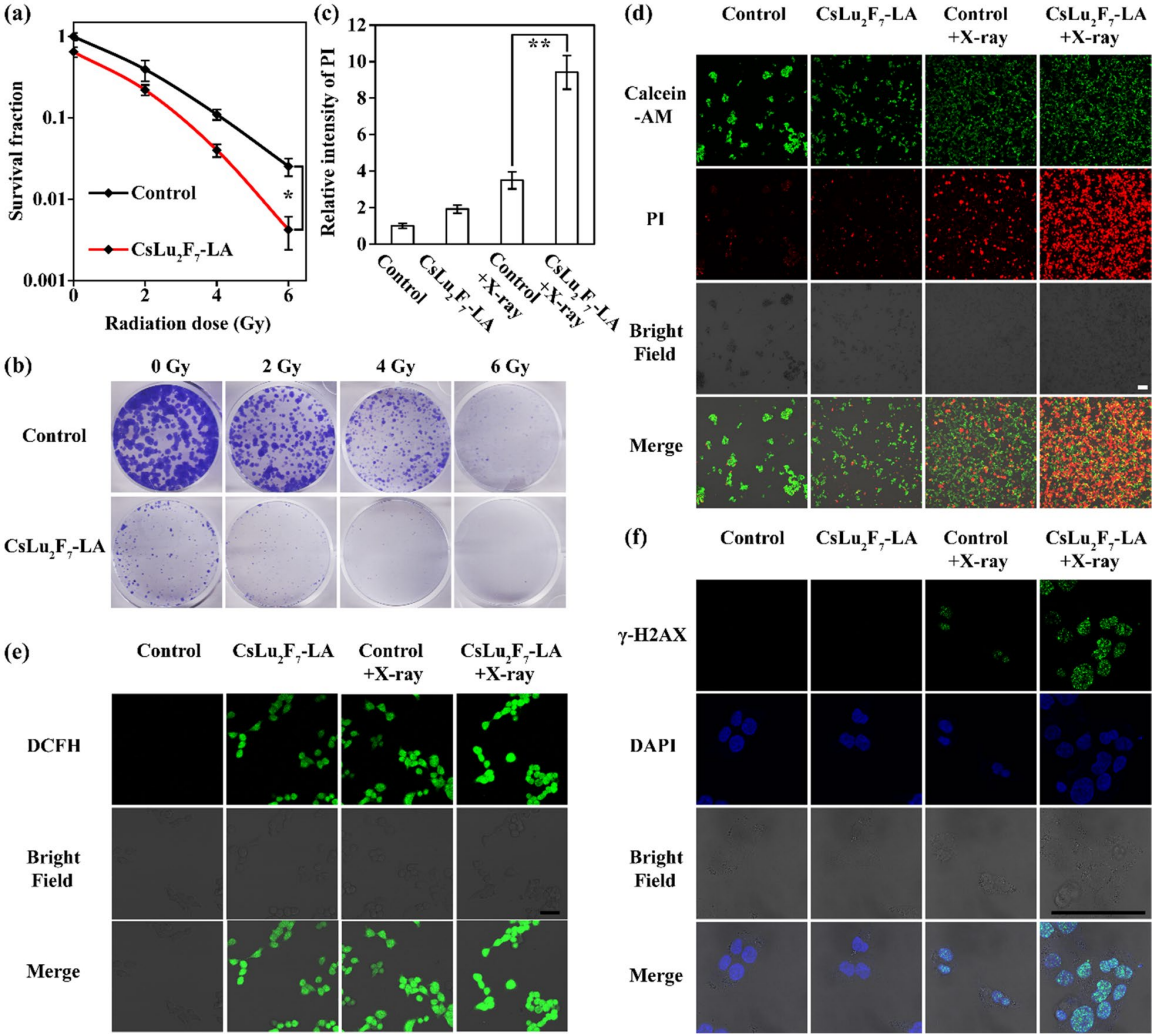
Experiments in vitro. **a** Survival fraction of cell colony formation assay (n = 3, mean ± SD, single asterisk indicates P < 0.05 according to Student’s two-tailed t-test). **b** Photographs of cell colony formation assay. **c** Relative intensity of PI in each group (n = 3, mean ± SD, two asterisks indicate P < 0.01 according to Student’s two-tailed t-test). **d** Calcein-AM and PI staining of each group. **e** The yield of ·OH of each group measured by 2,7-Dichlorodi-hydrofluorescein diacetate (DCFH-DA) staining. **f** Cell γ-H2AX staining of each group. Scale bar: 100 μm

Having proved that CsLu_2_F_7_-LA can slow down glycolysis, in this section, we explore the enhancement of CsLu_2_F_7_-LA to RT in vitro. Cell colony formation assay indicates that single CsLu_2_F_7_-LA can decrease the proliferation of cancer cells because of the deceleration of metabolism as mentioned above (Fig. 4a, b). Meanwhile, CsLu_2_F_7_-LA can also enhance the proliferative damage of RT with the increase of radiation dose (Fig. 4a, b). The staining of prodium iodide (PI) show that the combination of CsLu_2_F_7_-LA and X-ray can induce the most cell death (Fig. 4c, d). Apoptotic analysis measured by flow cytometry presents similar results (Figure S8). The enhancement of RT can be attributed to two reasons. For one thing, CsLu_2_F_7_-LA containing high-Z atoms, which can deposit more X-rays and increase the yield of ROS compared with other groups (Fig. 4e). For another, CsLu_2_F_7_-LA can decelerate glycolysis of cancer cells, leading to decreased repairment of DNA damage. Then it is not surprising that the combination of CsLu_2_F_7_-LA and X-ray can induce the most DNA DSBs (Fig. 4f and Additional file 1: Fig. S9), which will induce serious proliferative injury and cell death. Hence, these data prove that CsLu_2_F_7_-LA can enhance the effect of RT in vitro.

### CsLu_2_F_7_-LA enhance the effect of RT in vivo

Encouraged by the experiments in vitro, we then verify the performance of CsLu_2_F_7_-LA in vivo. Firstly, the biological compatibility of CsLu_2_F_7_-LA is evaluated based on Kunming mice. As shown in Additional file 1: Fig. S10a, the body weights have no obvious differnces among the groups injected with different dosage (0 mg/kg, 20 mg/kg, 40 mg/kg and 80 mg/kg) of CsLu_2_F_7_-LA. The blood biochemical indexes including alanine transaminase (ALT), aspartate transaminase (AST), alkaline phosphatase (ALP), urea and creatinine (CRE) of different dosage of CsLu_2_F_7_-LA also exhibit few differences (Additional file 1: Fig. S10b–f). Additionally, H&E staining of major organs (heart, liver, spleen, lung, and kidney) of each group present no pathological and anomalous regions (Additional file: Fig. S11). Hence, CsLu_2_F_7_-LA present favorable biological compatibility to normal tissues even at a dosage of 80 mg/kg.

**Fig. 5:**
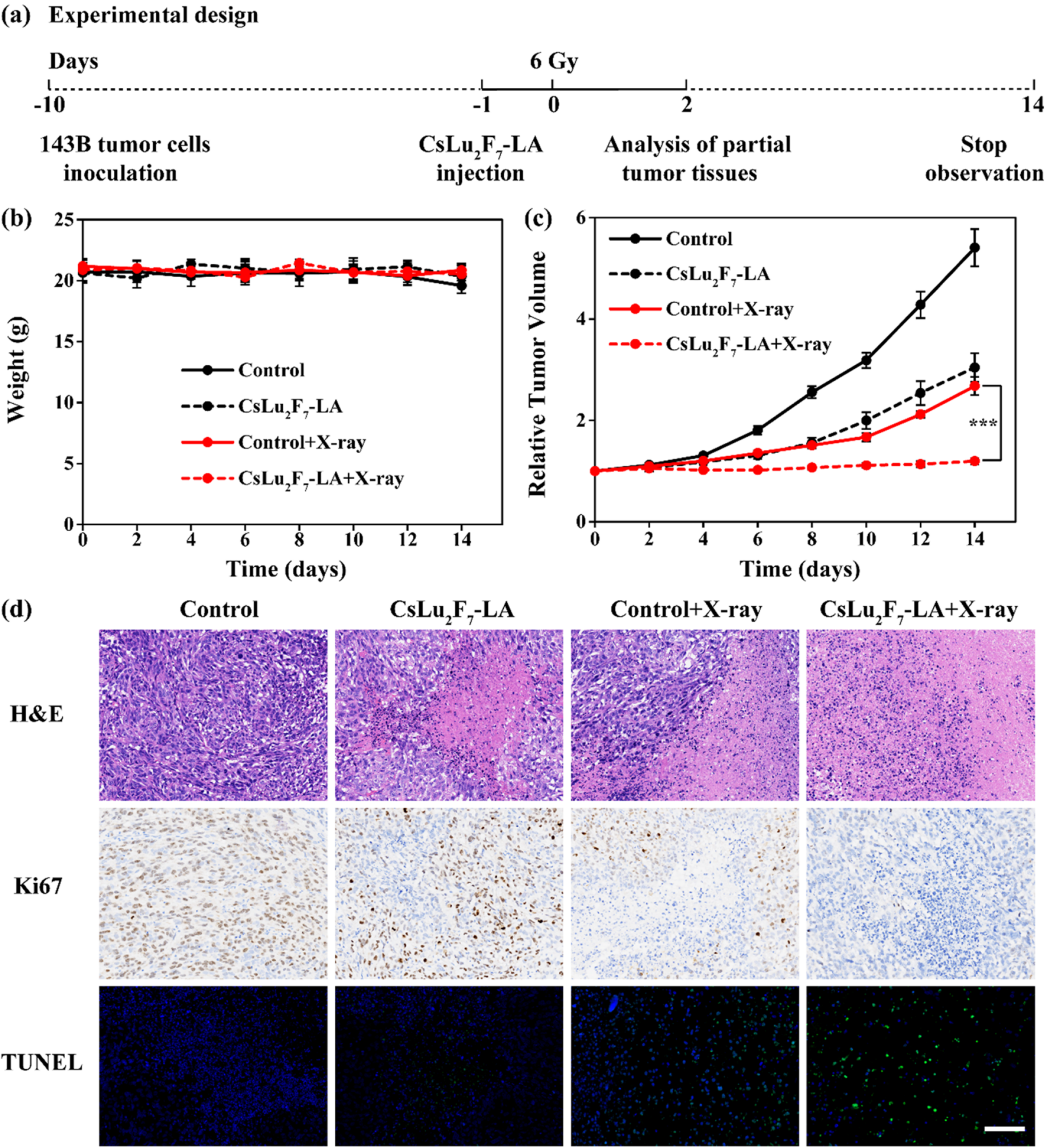
Experiments in vivo. **a** Illustration of the experimental design. **b** The weights of tumor-bearing mice of each group (6 Gy, n = 5, mean ± SD). **c** Relative tumor volume of each group (6 Gy, n = 5, mean ± SD, triple asterisks indicate P < 0.001 according to Student’s two tailed t-test). **d** H&E staining, Ki67 staining and TUNEL staining of tumor sections. Scale bar: 100 μm

Next, the curative effect of the combination of CsLu_2_F_7_-LA and X-ray is characterized. As shown in Fig. 5a, this experiment is based on 143B tumor-bearing mice. These mice are divided into four groups including Control group, CsLu_2_F_7_-LA group, Control + X-ray group and CsLu_2_F_7_-LA group. During the observation period the body weights of each group exhibit few differences (Fig. 5b). The data of curative effect (Fig. 5c) show that single CsLu_2_F_7_-LA and single X-ray can both limit the progression of tumor compared with Control group, and the combination of CsLu_2_F_7_-LA and X-ray present the best tumor suppression effect. Hematoxylin and eosin (H&E) staining, Ki67 staining, and terminal-deoxynucleoitidyl transferase mediated nick end labeling (TUNEL) staining of tumor sections also show that the combination of CsLu_2_F_7_-LA and X-ray can induce the most apoptosis and necrosis (Fig. 5d). All these data prove that CsLu_2_F_7_-LA can enhance the effect of RT in vivo.

## Conclusions

In summary, we synthesize CsLu_2_F_7_-LA for increasing the curative effect of RT. The contained Lu and Cs atoms can deposit much more X-rays in the process of RT to generate higher amount of ·OH. Meanwhile, CsLu_2_F_7_-LA can target MCT and hinder the transportation of LA, which lead to decreased glycolysis and DNA damage repair. As a result, the cancer cells will suffer from serious DNA DSBs and the curative effect of RT will be enhanced. All the experiments in vitro and in vivo prove the favorable performance of CsLu_2_F_7_-LA as radiosensitizers by disturbing glycolysis. RT is suitable for weak and elderly cancer patients because of the short treatment process and little invasive damage. However, radiation resistance is a bottleneck problem that limits the effect of RT. Generally, the specific metabolism of cancer cells supports themselves with proliferation and treatment resistance [45, 46]. In this research, we disturb glycolysis through LA accumulation to increase the effect of RT. More metabolic targets such as glutamine metabolism, pentose phosphate pathway, and tricarboxylic acid cycle can be explored in the future.

## Supplementary Information

**Additional file 1:** **Figure S1. **Hydrodynamic radius of CsLu_2_F_7_-OA measured by dynamic light scattering (DLS). **Figure S2. **FTIR spectra of CsLu_2_F_7_-OA and CsLu_2_F_7_-LA. **Figure S3. **Hydrodynamic size of CsLu_2_F_7_-LA dispersed in DI water, saline and DMEM at 1st day, 7th day and 14th day.** Figure S4. **Cs 3d, Lu 4d and F 1s X-ray photoelectron spectroscopy (XPS) spectrum. **Figure S5. **The yield of ·OH in solutions upon irradiation of 0 Gy, 5 Gy, 10 Gy, 15 Gy and 20 Gy X-rays (n = 5, mean ± SD). Four asterisks indicate P < 0.0001 according to Student’s two-tailed t-test. **Figure S6. **Relative release of Lu atoms of CsLu_2_F_7_-LA in saline (n = 3, mean ± SD). **Figure S7. **Cytophagy of CsLu_2_F_7_-LA (50 ppm). CsLu_2_F_7_-LA were decorated with FITC. Scale bar: 100 μm. **Figure S8. **Apoptotic analysis measured by flow cytometry. **Figure S9. **Relative intensity of γ-H2AX in each group (n = 3, mean ± SD). **Figure S10.** Biological compatibility evaluation of CsLu_2_F_7_-LA. **Figure S11.** H&E staining of major organs (heart, liver, spleen, lung, kidney) 30 days post intravenous injection of different dosage of CsLu_2_F_7_-LA. Scale bar: 100 μm.

## Data Availability

All study data are included in this article.
