# Supplementary material for: Lactic acid modified rare earth-based nanomaterials for enhanced radiation therapy by disturbing the glycolysis
Source: J Nanobiotechnology. 2022 Nov 19;20:490. doi: 10.1186/s12951-022-01694-1 (PMC9675198; doi:10.1186/s12951-022-01694-1)
Supplement: Supplementary file 1 — Additional file 1: Figure S1. Hydrodynamic radius of CsLu2F7-OA measured by dynamic light scattering (DLS). Figure S2. FTIR spectra of CsLu2F7-OA and CsLu2F7-LA. Figure S3. Hydrodynamic size of CsLu2F7-LA dispersed in DI water, saline and DMEM at 1st day, 7th day and 14th day. Figure S4. Cs 3d, Lu 4d and F 1s X-ray photoelectron spectroscopy (XPS) spectrum. Figure S5. The yield of ·OH in solutions upon irradiation of 0 Gy, 5 Gy, 10 Gy, 15 Gy and 20 Gy X-rays (n = 5, mean ± SD). Four asterisks indicate P < 0.0001 according to Student’s two-tailed t-test. Figure S6. Relative release of Lu atoms of CsLu2F7-LA in saline (n = 3, mean ± SD). Figure S7. Cytophagy of CsLu2F7-LA (50 ppm). CsLu2F7-LA were decorated with FITC. Scale bar: 100 μm. Figure S8. Apoptotic analysis measured by flow cytometry. Figure S9. Relative intensity of γ-H2AX in each group (n = 3, mean ± SD). Figure S10. Biological compatibility evaluation of CsLu2F7-LA. Figure S11. H&E staining of major organs (heart, liver, spleen, lung, kidney) 30 days post intravenous injection of different dosage of CsLu2F7-LA. Scale bar: 100 μm. [file 12951_2022_1694_MOESM1_ESM.docx]

Additional file 1

Lactic acid modified rare earth-based nanomaterials for enhanced radiation therapy by disturbing the glycolysis

Hu Liu^1,2^, Han Wang^3^*, Dalong Ni^3^*, Youjia Xu^1,2^*

^1^ *Department of Orthopedics, The Second Affiliated Hospital of Soochow University, Suzhou 215004, Jiangsu Province, China*

^2^ *Department of Orthopedics, Yancheng Third People’s Hospital, Yancheng 224001, Jiangsu Province, China*

^3^ *Department of Orthopaedics, Shanghai Key Laboratory for Prevention and Treatment of Bone and Joint Diseases, Shanghai Institute of Traumatology and Orthopaedics, Ruijin Hospital, Shanghai Jiao Tong University School of Medicine, Shanghai 200025, China*

*Corresponding: wanghan2021@sjtu.edu.cn; ndl12353@rjh.com.cn; xuyoujia@suda.edu.cn


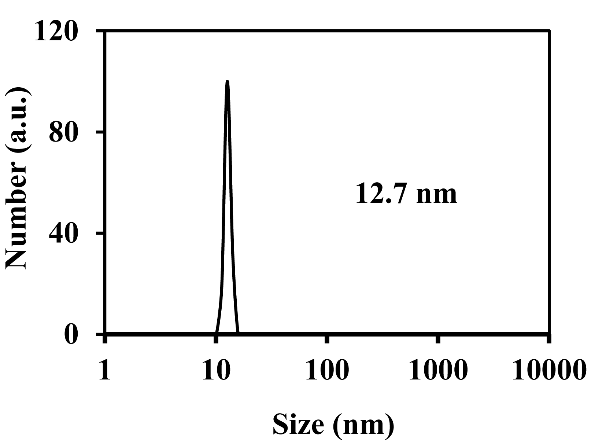


**Figure S1.** Hydrodynamic radius of CsLu_2_F_7_-OA measured by dynamic light scattering (DLS).


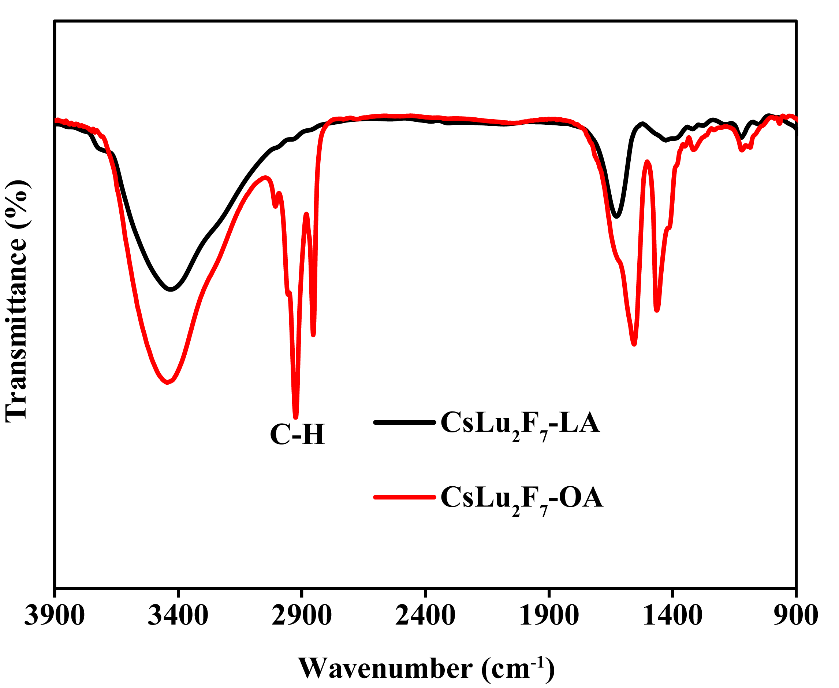


**Figure S2.** FTIR spectra of CsLu_2_F_7_-OA and CsLu_2_F_7_-LA.


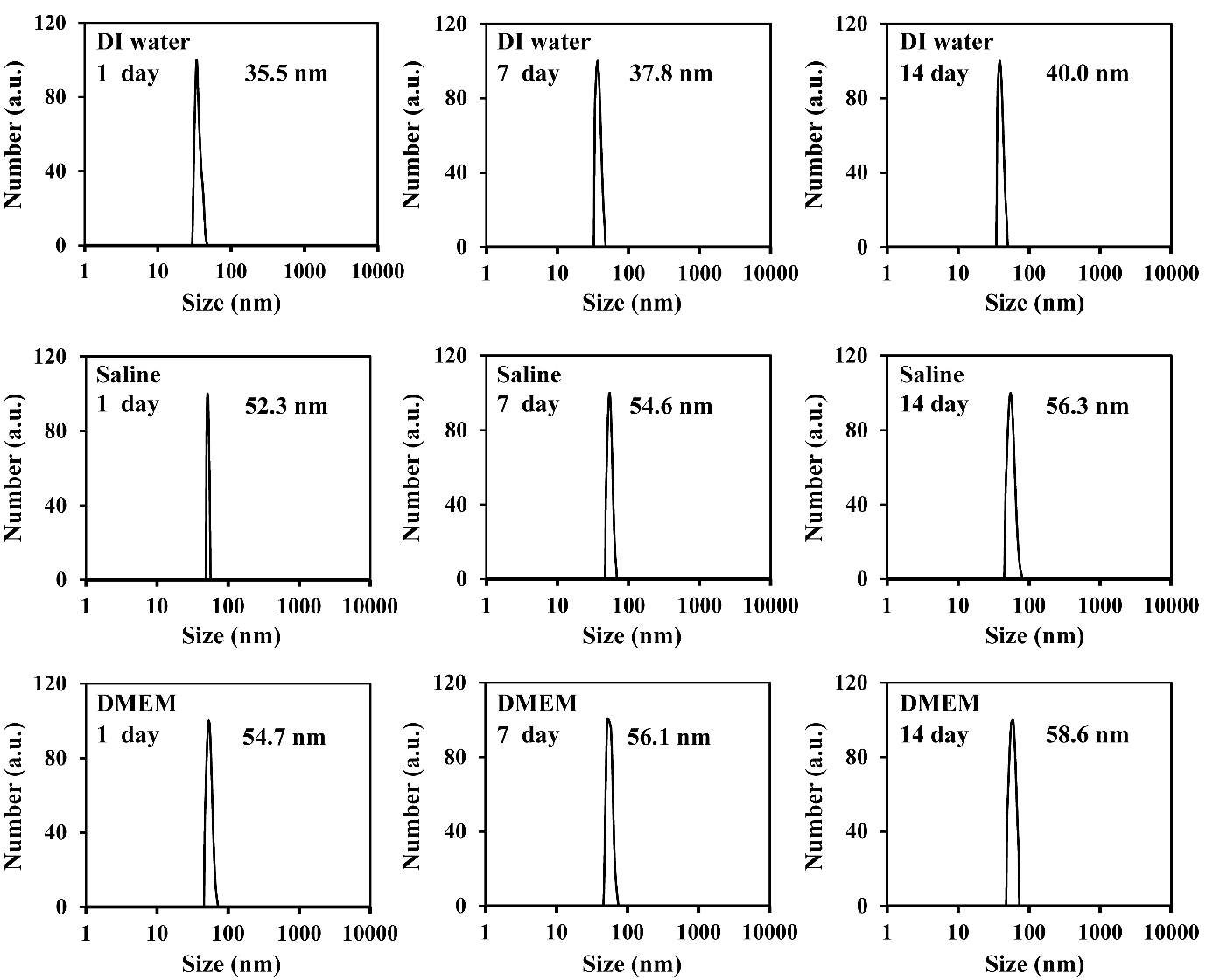


**Figure S3.** Hydrodynamic size of CsLu_2_F_7_-LA dispersed in DI water, saline and DMEM at 1st day, 7th day and 14th day.


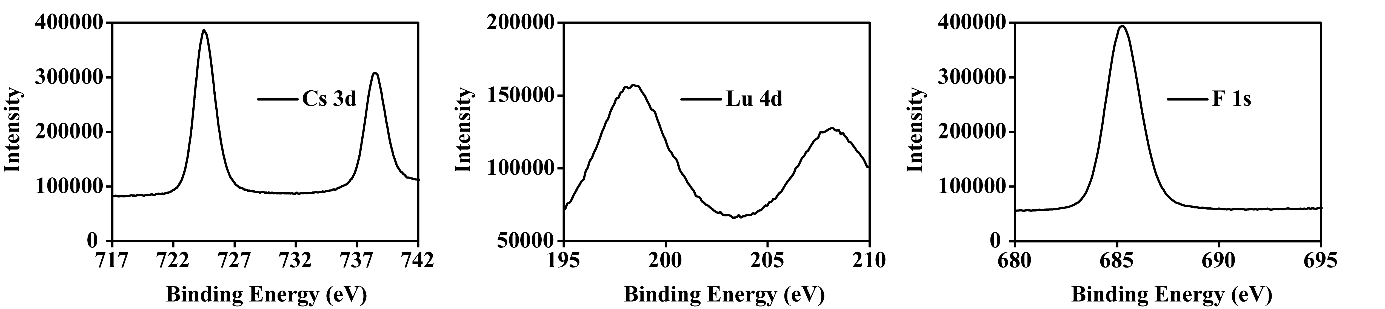


**Figure S4.** Cs 3d, Lu 4d and F 1s X-ray photoelectron spectroscopy (XPS) spectrum.


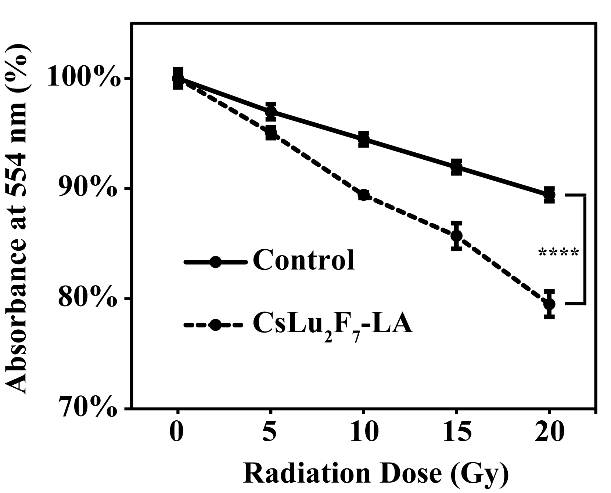


**Figure S5.** The yield of ·OH in solutions upon irradiation of 0 Gy, 5 Gy, 10 Gy, 15 Gy and 20 Gy X-rays (n = 5, mean ± SD). Four asterisks indicate P < 0.0001 according to Student’s two-tailed t-test.


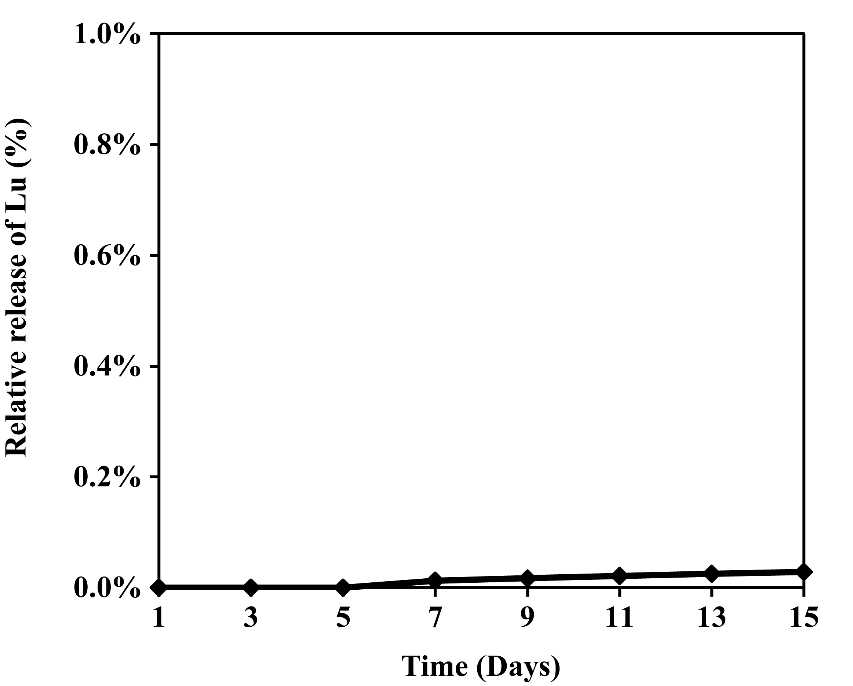


**Figure S6.** Relative release of Lu atoms of CsLu_2_F_7_-LA in saline (n = 3, mean ± SD).


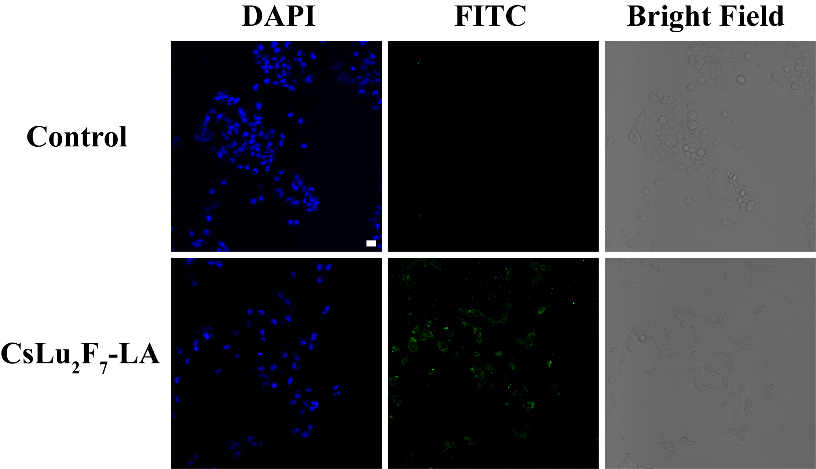


**Figure S7.** Cytophagy of CsLu_2_F_7_-LA (50 ppm). CsLu_2_F_7_-LA were decorated with FITC. Scale bar: 100 μm.

**
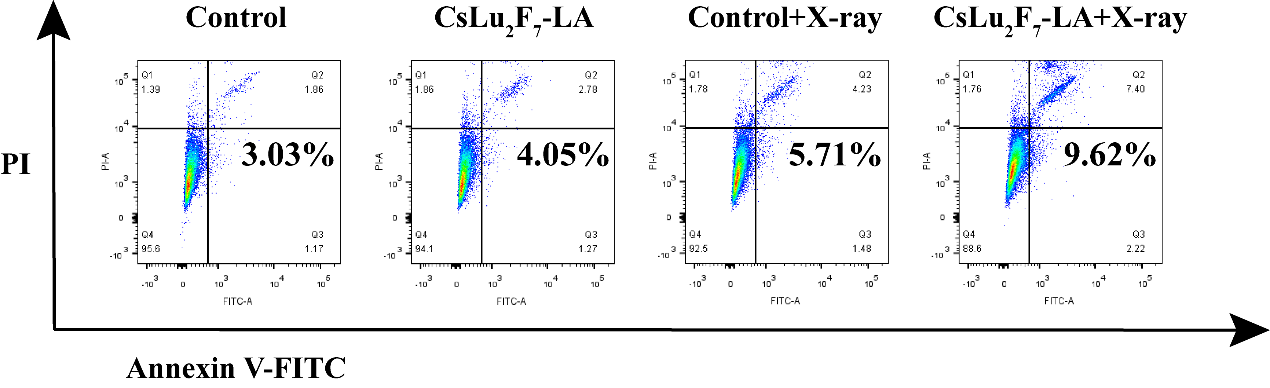
**

**Figure S8.** Apoptotic analysis measured by flow cytometry.

**
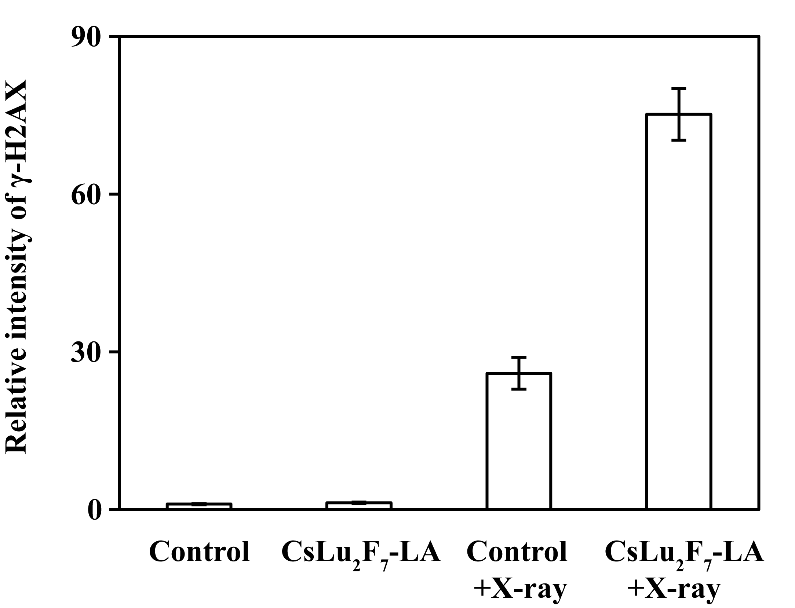
**

**Figure S9.** Relative intensity of γ-H2AX in each group (n = 3, mean ± SD).

**
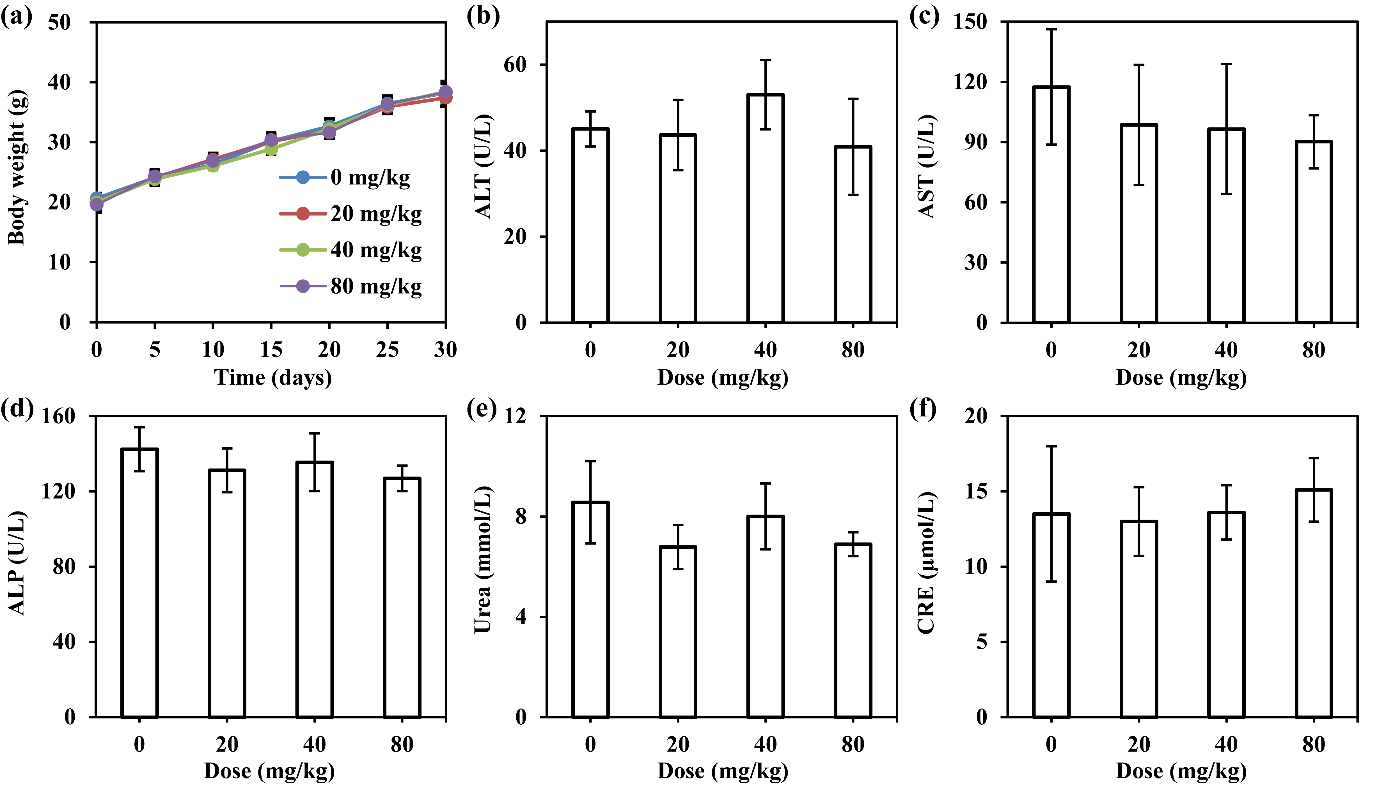
**

**Figure S10.** Biological compatibility evaluation of CsLu_2_F_7_-LA. a) The weights of Kunming mice post intravenous injection of different dosage of CsLu_2_F_7_-LA (n = 5, mean ± SD). Blood biochemical indexes including b) ALT, c) AST, d) ALP, e) urea and f) CRE 30 days post intravenous injection of different dosage of CsLu_2_F_7_-LA (n = 5, mean ± SD).


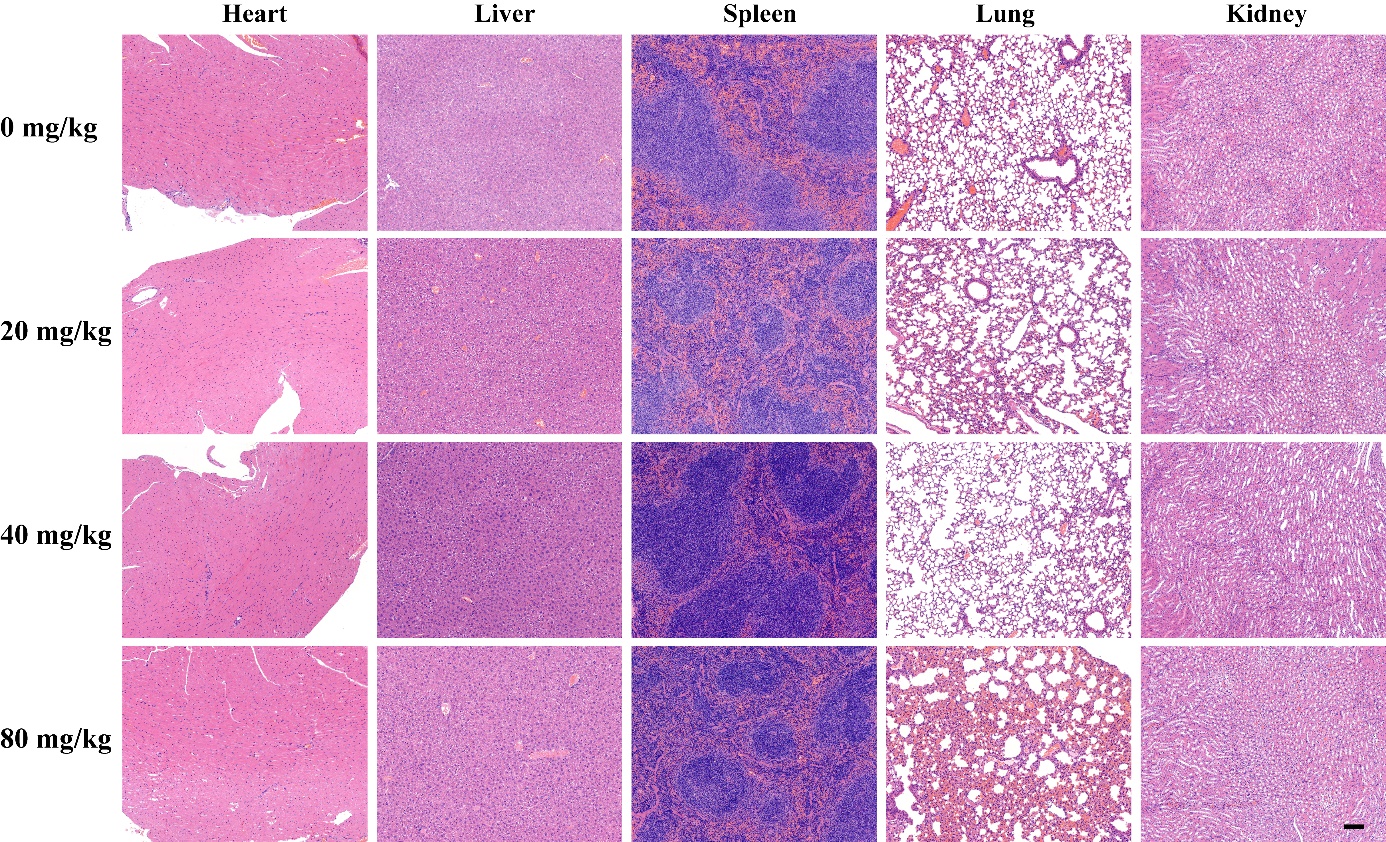


**Figure S11.** H&E staining of major organs (heart, liver, spleen, lung, kidney) 30 days post intravenous injection of different dosage of CsLu_2_F_7_-LA. Scale bar: 100 μm.
